# Topic-specific living databases of clinical trials: A scoping review of public databases

**DOI:** 10.1177/17407745251400635

**Published:** 2026-01-05

**Authors:** Kim Boesen, Lars G Hemkens, Perrine Janiaud, Julian Hirt

**Affiliations:** 1 Pragmatic Evidence Lab, Research Center for Clinical Neuroimmunology and Neuroscience (RC2NB), University Hospital Basel and University of Basel, Basel, Switzerland; 2 Meta-Research Innovation Center at Stanford (METRICS), Stanford University, Stanford, CA, USA; 3 Department of Health, Eastern Switzerland University of Applied Sciences, St. Gallen, Switzerland; 4 Institute of Health and Nursing Science, Medical Faculty, Martin Luther University Halle-Wittenberg, Halle (Saale), Germany

**Keywords:** Databases, randomised controlled trial, controlled clinical trials, systematic reviews, evidence mapping, literature search

## Abstract

**Introduction:**

Conducting systematic reviews of clinical trials is time-consuming and resource-intensive. One potential solution is to design databases that are continuously and automatically populated with clinical trial data from harmonised and structured datasets. This scoping review aimed to identify and map publicly available, continuously updated, topic-specific databases of clinical trials.

**Methods:**

We systematically searched PubMed, Embase, the preprint servers medRxiv, arXiv, Open Science Framework, and Google. We characterised each database using seven predefined features (access model, database type, data input sources, retrieval methods, data-extraction methods, trial presentation, and export options) and narratively summarised the results.

**Results:**

We identified 14 continuously updated databases of clinical trials, seven related to COVID-19 (initiated in 2020) and seven non-COVID-19 databases (initiated as early as in 2009). All databases, except one, were publicly funded and accessible without restrictions. Most relied on traditional methods used in static article-based systematic reviews sourcing data from journal publications and trial registries. The COVID-19 databases and some non-COVID-19 databases implemented semi-automated features of data import, which combined automated and manual data curation, whereas the non-COVID-19 databases mainly relied on manual workflows. Most reported information was metadata, such as author names, years of publication, and link to publication or trial registry. Only two databases included trial appraisal information (such as risk of bias assessments). Six databases reported aggregate group-level results, but only one database provided individual participant data on request.

**Discussion:**

Continuously updated topic-specific databases of clinical trials remain limited in number, and existing initiatives mainly employ traditional static systematic review methodologies. A key barrier to developing truly living platforms is the lack of accessible, machine-readable, and standardised clinical trial data.

## Introduction

The number of published clinical trials and systematic reviews increased tremendously over the past decades.^1,2^ It is difficult, if not impossible, for any clinician or researcher to stay up to date. Traditional systematic reviews are time-consuming and costly due to the efforts required to manually search databases, screen titles, abstracts, and full-text articles and data extraction.^3^ As a result, systematic reviews are often outdated and potentially even misleading by the time of publication.^4–7^

To address the limitations of traditional systematic reviews, the concept of ‘living systematic reviews’ was popularised. There is no universally accepted definition but living reviews are generally understood to be continuously updated (systematic) reviews.^8–11^ Living systematic reviews should mitigate delays and reduce the risk of missing important evidence. The COVID-19 pandemic accelerated the development of such ‘living’ projects reflecting the rapidly evolving evidence landscape. These include the COVID-19 trial tracker,^12^ the NMA-COVID Project,^13^ and WHO’s Living Guideline.^14^

Text mining and machine-learning assisted tools have been developed to automate parts of the review process and reduce resource demands^15–18^ focusing mainly on literature screening, retrieving records, and extracting data from publications.^19,20^ Whether existing living projects and topic-specific trial databases fully leverage advanced technologies to ensure seamless and – ideally fully automated – flow of clinical trial data into structured databases remains unclear.

We aimed to systematically identify publicly available, continuously updated, topic-specific clinical trial databases of clinical trials to characterise their basic functionalities, infrastructure, and user interface. This scoping review also served as a primer for designing a continuously updated database focused on cancer immunotherapies.^21^

## Methods

We conducted a scoping review and report it according to the Preferred Reporting Items for Systematic reviews and Meta-Analyses statement for Scoping Reviews (PRISMA-ScR) checklist.^22^ Due to the project’s exploratory nature, we did not initially register a protocol for this review.

### Eligibility criteria

We included any publicly available continuously updated database retrieving clinical trials (participants allocated to two or more groups); irrespective of retrieval method (e.g. manually, automated, or a combination), and input source (e.g. databases of published literature, trial registries, or both). We did not define a minimum update frequency threshold for the included databases. We considered topic-specific databases only, that is, exclusive to certain conditions, interventions, or indication, such as a COVID-19. Databases including other research designs in addition to clinical trials, such as observational studies, were considered eligible. We did not include broad, topic-unspecific trial databases, such as clinical trial registries. We applied no restrictions on language, release date, intended use (i.e. for research or clinical decision-making), or database status (i.e. live or archived).

### Information sources, search strategy, and selection of sources of evidence

We employed a two-step systematic search strategy. We did ‘cold searches’ of various resources to identify key words and references to subsequently build systematic database search strategies.

Step 1: Cold search – One author (KB) searched PubMed (including using the ‘similar articles’ function), preprint servers medRxiv and ArXiv, Open Science Framework, and Google (first five pages for each search) informed by key search terms and databases known to us prior to this scoping review (S1 and S2, Appendix in the Supplementary Material).
Step 2: Systematic search – The PubMed and Embase/Elsevier databases were systematically searched by one author (JH). Two authors (KB, JH) designed a three-component search strategy containing keywords for ‘living’ (e.g. live, updated, and interactive), ‘library’ (e.g. database, platform, and repository), and study design (e.g. trial, and clinical study). See full search strategies and search dates in the Appendix, S3 and S4. The retrieved records were deduplicated using Citavi^23^ and imported into the screening tool Rayyan.^24^ One author (KB) screened titles, abstracts, and full texts and decided on inclusion in one step. Uncertainties were discussed with another author (PJ or JH) to reach a final decision.

### Data extraction and narrative summary

**Figure 1.**
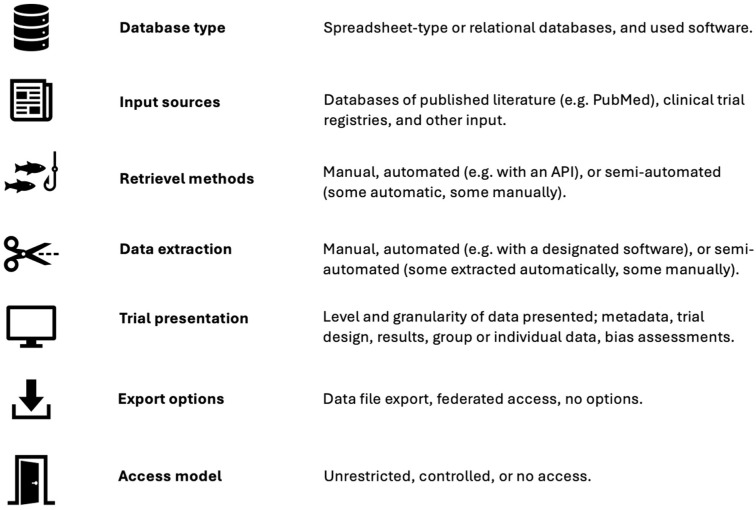
Data-extraction variables on characteristics of continuously updated clinical trial databases.

We narratively summarised the following seven database characteristics based on information available from the database websites and corresponding journal publications (Figure 1). One reviewer (KB) extracted information, and one reviewer (JH) double-checked and confirmed the extractions. Data was tabulated directly in Microsoft Word and screenshots to document the user interface of eligible databases.

### Database characteristics

1. Database type: What was the underlying database type and database-management software (IBM Ref)? Options include simple Excel spreadsheets or conventional relational databases,^25^ which may be managed with various database-management systems, like RedCap.^26^
2. Input sources: Which data input sources were searched? Databases of published literature like PubMed or Embase, trial registries, for example, ClinicalTrials.gov, or other sources.
3. Retrieval methods: How was the database populated? Manually, automated, for example, by application programming interfaces (APIs), or via community-based data submissions where researchers submit data to the database, like ClinicalTrials.gov or the nucleotide sequencing database GenBank.^27^
4. Data extraction: How was the trial information extracted and curated? Manually, fully automated with designated software tools,^15–17^ or a combination of machine-assisted manual curation, usually referred to as ‘semi-automated’.^28^
5. Trial presentation: What trial information were reported on the website? Meta data (e.g. title, author names, publication or trial registry information), detailed trial information (e.g. study design, sample size, treatment descriptions, funding), results (outcomes and effect estimates), type of results (aggregate group level or individual participant data), and trial appraisal (e.g. risk of bias assessments, limitations in the trial design).
6. Export options: What were the options for downloading and reusing data? Free download of data (e.g. as CSV or Excel files), federated access^29^ (e.g. users can access the data remotely, usually in browser-based applications, but without options for downloading it to their own personal computers, such as Vivli.com),^30^ or no option for exporting data.
7. Access model: How was the database content accessed? Unrestricted access (i.e. data is freely available without any constraints), controlled access (e.g. users must submit requests and the provider grants or denies access), or a hybrid between the two.

## Results

### Search results

Our systematic database search returned 1707 hits (after deduplication); 15 were assessed in full-text (see reasons for exclusion in S6, Appendix in the Supplementary Material) and one database (TrialsResultsCenter^31,32^) was included. One database (Evidence Finder^33–35^) was found through other sources, nine databases (Cochrane COVID-19 Study Register,^36^ COVID-evidence,^37^ COVID TrialsTracker,^12^ COVID-NMA initiative,^13^ EPPI Centre Covid-19 Living Map of the Evidence,^38^ MetaEvidence breast cancer,^39^ MetaEvidence COVID,^40^ MetalO,^41^ and MetaPreg^42^) were known to us before our systematic search. Our cold searches yielded three eligible databases (Infectious Disease Data Observatory (IDDO),^43^ Worldwide Antimalarial Resistance Network (WWARN),^44–46^ and EvidenceMap^47–49^ (S5, Appendix in the Supplementary Material).

In total, we included 14 databases (Table 1), seven non-COVID-19 databases on youth mental health (Evidence Finder), pancreatic surgery (Evidence Map), cardiology and oncology trials (TrialResultsCenter), cancer (MetaEvidence breast cancer and MetalO), pregnancy (MetaPreg), and malaria (WWARN) and seven COVID-19 databases (Cochrane COVID-19 Study Register, COVID-evidence, COVID TrialsTracker, COVID-NMA initiative, EPPI Centre Covid-19 Living Map of the Evidence, IDDO, and MetaEvidence COVID).

**Table 1.** Included continuously updated field-specific clinical trials databases.

Name	Year	Field	Funding/responsible organisation	Access
Cochrane COVID-19 Study Register	2020–2024	COVID-19	Cochrane and the Federal Ministry of Education and Research of Germany (CEOsys project)	Fully open
COVID-evidence	2020–2022	COVID-19	Swiss National Science Foundation	Fully open
COVID NMA initiative	2020–2023	COVID-19	Centre for Research in Epidemiology and Statistics (CRESS) and Cochrane France	Fully open
COVID Trials Tracker	2020–2022	COVID-19	Centre for Evidence Based Medicine, Oxford University	Fully open
EPPI Centre Covid-19 Living Map of the Evidence	2019–2023	COVID-19	Evidence for Policy and Practice (EPPI) Centre and the UK National Institute for Health and Care Research (NIHR) Policy Research Programme Reviews Facility	Fully open
Evidence Finder	2010–2021	Youth mental health	Orygen Ltd^a^	Fully open
Evidence Map	2021–active	Pancreatic surgery	International Study Group for Pancreatic Surgery and Viatris^b^	Fully open
IDDO Infectious disease Data Observatory Living systematic review	2020–active	COVID-19	Coalition for Equitable ResearCh in Low-resource sEttings (CERCLE)^c^	Fully open
MetaEvidence breast cancer	Not stated	Breast cancer	EU Horizon 2020 Qualitop, Hospices Civils de Lyon, CNRS UMR5558 LBBE	Fully open
MetaEvidence COVID	2020–2022	COVID-19	Hospices Civils de Lyon	Fully open
MetalO	Not stated	Cancer	EU Horizon 2020 Qualitop, Hospices Civils de Lyon, CNRS UMR5558 LBBE	Fully open
MetaPreg	Not stated	Pregnancy	ANSM (agence Nationale de Sécurité du Médicament), Hospices Civils de Lyon and University Lyon-1 (LBBE)	Fully open
TrialResultsCenter	2009–2017	Cardiology and oncology	Hospices Civils de Lyon	Fully open
WWARN Worldwide Antimalarial Resistance Network Clinical Trials Publication Library	2011–active	Malaria	Coalition for Equitable ResearCh in Low-resource sEttings (CERCLE)^d^	Fully open

a An Australian charity devoted to mental health issues (https://www.orygen.org.au/).

b We could not find information on the international study group for pancreatic surgery including its funding. Viatris is a pharmaceutical company.

c An international research network currently funded by the German ministry of education and research.

d WWARN is one of IDDO’s projects; thus, we assume the funding is the same.

### Key findings

There were important trends and differences between the 14 identified databases indicating that the COVID-19 databases leveraged methods and technology to automatise several of the time-consuming tasks, whereas the non-COVID-19 databases largely replicated methods from traditional systematic reviews without integrating methods to streamline the workflow. While the COVID-19 databases were launched during the pandemic in 2020 (and all but one is no longer maintained), the oldest non-COVID-19 databases date back to 2009–2011. The database types were unclearly reported for most databases, and the input sources were generally similar. The most important differences between COVID-19 and non-COVID-19 databases pertain to the retrieval and data-extraction methods. The non-COVID-19 databases largely used manual retrieval methods (four manual, one semi-automated, two unreported) and data extraction (five manual, two unreported); meanwhile the COVID-19 databases were more advanced, and five databases used semi-automated retrieval and data-extraction methods and only two used manual methods. All databases reported basic meta-data information (including title, authors, and links to publications/registry entries), but only few databases reported more granular results and bias assessments.

### Database characteristics – non-COVID-19 databases

The TrialResultsCenter was active from 2009 to 2017 and is no longer updated. EvidenceFinder was released in 2010 with the last update in July 2021, though its current maintenance status is uncertain. WWARN was released in 2011, but its maintenance status is also unclear; the last trial in their Data Inventory^44^ dates from 2018, but their trial summary^43^ says it is current until 2022. EvidenceMap was launched in 2021 with the most recent update on July 26, 2023. The release dates were not specified for three databases (MetaEvidence breast cancer, MetalO, and MetaPreg). Six of the seven non-COVID-19 databases were either publicly funded or funded by non-profit organisations while one (Evidence Map) was funded by a pharmaceutical company. All database websites were fully accessible with no restrictions.

The databases primarily searched common sources, such as PubMed/MEDLINE, Embase, and Web of Science, employing methods similar to those used in systematic reviews, including manual database searches, data extraction, and curation. EvidenceMap was built on a relational Microsoft SQL server database, and WWARN used a proprietary data management tool (RedCap). The database type for the remaining five was not specified.

Regarding data availability, five databases (EvidenceMap, MetaEvidence breast cancer, MetalO, MetaPreg, and WWARN) made their data readily available for download. One database (TrialResultsCenter) provided data upon request, and another (EvidenceFinder) did not specify download options (see Table 2).

**Table 2.** Database characteristics (non-COVID 19).

Name	Database type	Input sources	Retrieval methods (frequency)	Data extraction	Trial presentation	Export
Evidence Finder	Not described	MEDLINE, Embase, PsychINFO, Cochrane Library	Manually (yearly)	Manual	Metadata, trial information	Not mentioned
Evidence Map	Relational database (Windows Form .NET management system)	PubMed, CENTRAL, Web of Science	Manually (frequency not described)	Manual	Metadata, trial appraisal	Excel download
MetaEvidence Breast cancer	Not described^a^	Not described^a^	Not described^a^	Not described^a^	Metadata, trial information, trial appraisal, results (group level)	No option
MetalO	Not described^a^	Not described^a^	Not described^a^	Not described^a^	Metadata, trial information, trial appraisal, results (group level)	No option
MetaPreg	Not described (“Proprietary meta-analysis platform”)	PubMed, Scopus, Web of Science, 17 preprint servers	Semi-automated (daily) (“using automatic software robots”)	Manual	Metadata, trial information, trial appraisal, results (group level)	No option
Trial Results Centre	Not described	MEDLINE, Embase, CENTRAL, ClinicalTrials.gov, ISRCTN, ICTRP	Manually (weekly)	Manual	Metadata, trial information, results (group level)	On request
WWARN	Relational database (RedCap)	PubMed, Embase, Web of Science	Manually (every 6 months)	Manual	Metadata	Excel download

a There are no separate methods’ description for these platforms. They link to the metaEvidence COVID methods.

All databases included basic metadata information, such as title, author names, and links to publication or trial registries. Three databases (MetaEvidence breast cancer, MetalO, and MetaPreg) reported aggregated group-level results and risk of bias assessments. One (TrialsResultsCenter) reported aggregated group-level results while two databases (Evidence Finder and TrialsResultsCenter) reported some trial information. EvidenceMap uniquely reported trial appraisal as a risk of bias assessment (Table 2; S7, Appendix in the Supplementary Material).

### Database characteristics – COVID-19 databases

The seven COVID-19 databases were initiated shortly after the SARS-CoV-2 outbreak in early 2020. By January 2024, the last of these databases, the Cochrane COVID-19 Study Register had ceased updates. All databases were publicly funded or supported by non-profit institutions and were openly accessible to the public without restrictions.

Three of the seven COVID-19 databases (Cochrane COVID-19 Study Register, EPPI Centre Covid-19 Living Map of the Evidence, and MetaEvidence COVID) primarily searched common sources, including databases of published reports such as PubMed/MEDLINE and Embase. In addition, six of the seven databases also searched one or more trial registries, broadening the scope of evidence retrieval.

Five databases (Cochrane COVID-19 Study Register, COVID-evidence, COVID TrialsTracker, EPPI Centre Covid-19 Living Map of the Evidence, and Meta-evidence) used semi-automated methods for study retrieval and data extraction, particularly for trial registry records.

In contrast, two databases (COVID NMA Initiative and IDDO) relied solely on manual data retrieval and extraction.

Two databases (IDDO and COVID-evidence) used proprietary database-management tools (RedCap and Directus). The database-management systems for the other five were not specified.

In terms of data availability, six databases (Cochrane COVID-19 Study Register, COVID-evidence, COVID TrialsTracker, EPPI Centre Covid-19 Living Map of the Evidence, MetaEvidence COVID, and IDDO) provided data for direct download, while one database (COVID NMA initiative) made data available upon request (Table 3).

**Table 3.** Database characteristics (COVID-19 specific).

Name	Database type	Input sources	Retrieval methods (frequency)	Data extraction	Trial presentation	Export
Cochrane COVID-19 Study Register	Not described	PubMed, Embase, Cochrane Central Register of Controlled Trials (CENTRAL), ClinicalTrials.gov, WHO International Clinical Trials Registry Platform (ICTRP), medRxiv, Retraction Watch	Semi-automated (daily to monthly)	Semi-automated	Metadata, trial information	CSV (no longer available)
COVID-evidence	Relational database (managed by Directus)	ClinicalTrials.gov, ICTRP, LOVE platform	Semi-automated (weekly)	Semi-automated	Metadata, trial information	Excel and CSV download
COVID NMA initiative	Not described	ICTRP, LOVE, Cochrane COVID-19 register	Manual (monthly)	Manual	Metadata, trial information, results (group level)	On request
COVID TrialsTracker	Not described	ICTRP	Semi-automated (weekly)	Semi-automated	Metadata, trial information	Excel and CSV download
EPPI Centre Covid-19 Living Map of the Evidence	Not described	Microsoft Academic Graph (MAG) dataset, OpenAlex (since 2020; before MEDLINE and Embase)	Semi-automated (bi-weekly to monthly)	Semi-automated	Metadata, trial information	Excel, Citations (.txt), and Research Information System (RIS)
IDDO	Relational database (RedCap)	ICTRP	Manual (monthly)	Manual	Metadata, trial information, individual trial participant data (on request)	Excel download
MetaEvidence COVID	Not described	PubMed, LitCovid, Scopus, Web of Science, ClinicalTrials.gov, WHO ICTRP, 17 preprint servers	Semi-automated (daily)	Semi-automated^a^	Metadata, trial information, results (group level), trial appraisal	Excel download

a Abstracts of studies identified in the above search were examined by a biocurator and an automatic classification algorithm. Each abstract is analyzed by this automatic classification algorithm that determines an index of relevance of the abstract for meta-analysis. This automatic classification algorithm is based on naïve Bayes classifier.

All databases reported basic metadata, including trial information such as study type or design, sample size or expected starting date (extracted from ClinicalTrials.gov). Two (Covid NMA Initiative and MetaEvidence COVID) also reported aggregate group-level results, and while MetaEvidence COVID uniquely included trial appraisal through risk of bias assessments (Table 3; S7, Appendix in the Supplementary Material).

## Discussion

### Summary of evidence

We identified 14 clinical trial databases, of which seven were related to COVID-19. The databases differed significantly in their data retrieval methods and maintenance approaches. COVID-19 databases predominantly used semi-automated methods to retrieve and extract data, primarily from clinical trial registries. In contrast, non-COVID databases mainly relied on manual methods inspired by systematic reviews, which are typically time-consuming and difficult to maintain. While all databases mainly provided basic metadata, the availability of curated trial information, results, or trial appraisal was sparse. Only one database (IDDO) explicitly stated to share individual participant data (on request). This highlights a gap in data sharing and transparency for most databases.

### Findings in context

Our results highlight a significant gap in the availability of continuously updated, topic-specific clinical trial databases. Existing databases largely borrow methods and technology from static article-based systematic reviews requiring manual retrieval, data extraction, and curation from unstructured journal articles. In addition, data captured or exported from clinical trial registries are often not easily integrated into structured databases, further compounding the issue.

Despite the development of various databases, none of the 14 platforms effectively addressed the fundamental problem of automatically integrating structured, machine-readable datasets from diverse sources. Currently, there are no publicly available standardised datasets that could seamlessly feed into such databases.

The first requirement to enable such continuously updated databases of clinical trial data is to have all trial data available in machine-readable and harmonised formats.^50^ The idea of such machine-readable repository of RCTs dates back over 25 years back to the Global Trial Bank which was envisioned to go beyond trial registration and provide trial results in a computable format.^51–54^ Although the Global Trial Bank never materialised, the 2004 policy by the International Committee for Journal Medical Editors requiring trial registration as a condition for publication marked an important step.^55^ This was followed by requirements to post summary results on ClinicalTrials.gov (from 2007) and the EU Clinical Trials Register (from 2012).^56^

Some researchers have argued that clinical trial data should be made available in a structured dataset, such as the Common Technical Document format used for commercial drug applications.^57^ Others have proposed that evidence synthesis, like systematic reviews, should rely primarily on clinical trial registries as the main data source, rather the traditional publication-based sources, to enable more timely and comprehensive synthesis.^58^ The concept of the Global Trial Bank and of continuously updated, clinical trial databases lies at the intersection of these two ideas.

### Future directions or emerging solutions

**Figure 2.**
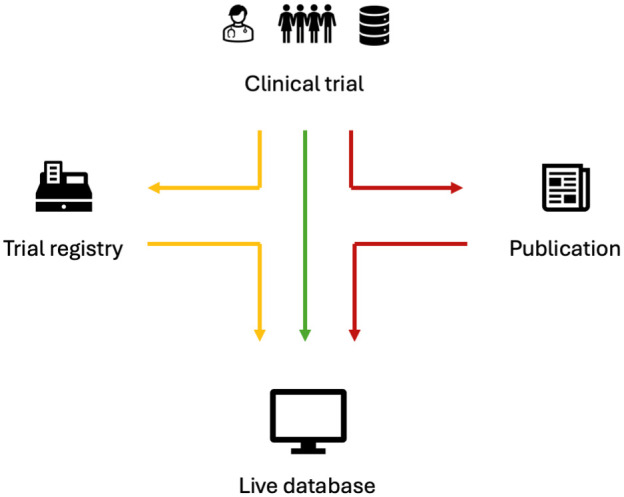
Pathways from clinical trial data into live topic-specific databases. Green path: sharing and integrating data directly from individual clinical trials. Yellow path: sharing and reusing trial data from the clinical trial registries. Red path: accessing and reusing data from journal publications.

To sum up, the challenges to create living, continuously updated, topic-specific, trial databases can be addressed on several levels taking into consideration the life cycle of an individual clinical trial; (1) data may be captured and integrated directly from a clinical trial into a live database (green path); (2) data may be shared to clinical trial registries and reused in a uniform standardised format (yellow path); (3) data may be accessed and reused from traditional journal publications (red path) (Figure 2).

1. Capturing and reusing data directly from the clinical trials in standardised have been tested in initiatives, such as FDA Critical Path Initiative,^59^ which has developed databases on Alzheimer’s,^60,61^ Multiple Sclerosis,^62^ Parkinson’s,^63^ and tuberculosis^64^ clinical trial data. However, these resources remain private or public-private and are not universally accessible. The prevalence and accessibility of similar data platforms are not well documented, indicating a need for more transparent and accessible clinical trial data infrastructure.
2. There are several reasons why clinical trial registries in their current shape are challenging as data sources for creating topic-specific trial databases. These limitations include a lack of data harmonisation, data quality issues, and data export options (see Table 4 for details).
3. One potential tool to automatise data extraction from journal publications is TrialStreamer, a mega-collection of PubMed-indexed clinical trials (n = 852,723 as of December 2023), which includes annotated PICO information (population, intervention, comparator, outcome).^65^ Trial Streamer utilises a blend of machine-learning and crowd-sourced manual extraction to gather trial data. While useful for meta-epidemiological studies, it lacks the granularity required for clinical applications or guideline development.

**Table 4.** Challenges in using trial registries for topic-specific databases.

**Lack of harmonisation**: The various trial registries do not harmonise database schemes or data format structures beyond the WHO Trial Registration Data Set.^64^ This is a high-level checklist only, which do not specify detailed outcome measures or results reporting. **Data quality issues**: Our initial experience with the CIEL database (using the ClinicalTrials.gov dataset made available from the Aggregate Analysis of ClinicalTrials.gov (AACT) database via https://ctti-clinicaltrials.org/) indicates that data entry errors are common, leading to misplaced or inaccurate data within registry fields.^30^ This significantly limits the utility of machine-readable datasets, e.g., those accessible via APIs like ClinicalTrials.gov, without extensive data cleaning and validation. Thus, imported data needs a substantial amount of preparation and curation to become useful. **Limited Data Export**: Only a few trial registries offer data export options. For example, the European Union Clinical Trial Register does not support automated data access, making the process labour-intensive.

The importance of living evidence synthesis has been increasingly recognised, exemplified by the Wellcome Trust’s recent investment of GBP 45 million over 5 years to advance data infrastructure for dynamic evidence projects. This initiative reflects an acknowledgement of the need for more adaptable, continuously updated data ecosystems.^66^

### Limitations

Our scoping review has several limitations. We did not prespecify our methodology and register a protocol for this work. We believe that the impact is minimal considering the scope of the review and its exploratory nature without any quantitative analyses. We may have missed eligible databases during our searches due to their scarcity and lacking common terminology, and if such databases have not been described in related published articles, they may be invisible. It is likely that several ‘stealth’^67^ commercial solutions exist. An example of a public commercial solution is the LiveSLR programme,^68,69^ which is a software combining an annotated library of publications (called LiveRef) with a machine-assisted search/screening/ranking workflow, which creates an interactive live platform used in drug applications and health technology dossiers. Unfortunately, there is little transparency when it comes to such commercial solutions. We decided to not make detailed assessments of the included databases’ infrastructure, including granular descriptions of data retrieval and extraction from sources such as ClinicalTrials.gov. We did so mainly because these workflows were not transparently reported on websites or corresponding journal publications. Transparent and exhaustive reporting of the database infrastructure should be a priority because automation is the main methodological challenge. For example, the metaPreg platform reported to use ‘automatic software robots’ to screen databases, with no further specifications.^50^ There may be several reasons for vaguely describing the methodology, for example, if setups are updated and adjusted frequently. However, open and exhaustive descriptions of platform infrastructure, including how automation is attempted and achieved, is encouraged.

## Conclusions

This review is the first to systematically assess the characteristics and data management of continuously updated clinical trial databases. It provides insights into their design, limitations and highlights key technical and methodological challenges. We found only few topic-specific databases of clinical trials, which mainly relied on manual searches and manual data extraction. Such approaches are inefficient and time-consuming maintenance, compromising data timeliness and utility. Recent COVID-19 trial databases implemented semi-automated workflows, albeit confined to clinical trial registries. However, these workflows remain insufficient for integrating data from journal publications. A major challenge remains in automatically extracting and structuring trial data from publications. It seems timely to reconsider ideas such as the Global Trial Bank to centralise not only trial registration but also trial results reporting and data sharing. Facilitating the shift from article-based publications to a fully ‘digital’ machine-readable system of clinical trial data could enable true living evidence synthesis.

## Supplemental Material

sj-pdf-1-ctj-10.1177_17407745251400635 – Supplemental material for Topic-specific living databases of clinical trials: A scoping review of public databases Supplemental material, sj-pdf-1-ctj-10.1177_17407745251400635 for Topic-specific living databases of clinical trials: A scoping review of public databases by Kim Boesen, Lars G Hemkens, Perrine Janiaud and Julian Hirt in Clinical Trials

sj-pdf-2-ctj-10.1177_17407745251400635 – Supplemental material for Topic-specific living databases of clinical trials: A scoping review of public databases Supplemental material, sj-pdf-2-ctj-10.1177_17407745251400635 for Topic-specific living databases of clinical trials: A scoping review of public databases by Kim Boesen, Lars G Hemkens, Perrine Janiaud and Julian Hirt in Clinical Trials

## References

[1] Bastian H Glasziou P Chalmers I. Seventy-five trials and eleven systematic reviews a day: how will we ever keep up? PLoS Med 2010; 7: e1000326. 10.1371/journal.pmed.1000326 PMC2943439 20877712

[2] Andersen MZ Zeinert P Rosenberg J , et al. Comparative analysis of Cochrane and non-Cochrane reviews over three decades. Syst Rev 2024; 13: 120. 38698429 10.1186/s13643-024-02531-2 PMC11064235

[3] Borah R Brown AW Capers PL , et al. Analysis of the time and workers needed to conduct systematic reviews of medical interventions using data from the PROSPERO registry. BMJ Open 2017; 7: e012545. 10.1136/bmjopen-2016-012545 PMC5337708 28242767

[4] Tricco AC Brehaut J Chen MH , et al. Following 411 Cochrane protocols to completion: a retrospective cohort study. PLoS One 2008; 3(11): e3684. 10.1371/journal.pone.0003684 PMC2577297 18997866

[5] Andersen MZ Gülen S Fonnes S , et al. Half of Cochrane reviews were published more than 2 years after the protocol. J Clin Epidemiol 2020; 124: 85–93. 32413390 10.1016/j.jclinepi.2020.05.011

[6] Bashir R Surian D Dunn AG. Time-to-update of systematic reviews relative to the availability of new evidence. Syst Rev 2018; 7: 195. 30447694 10.1186/s13643-018-0856-9 PMC6240262

[7] Beller EM Chen JKH Wang ULH , et al. Are systematic reviews up-to-date at the time of publication? Syst Rev 2013; 2: 36. 23714302 10.1186/2046-4053-2-36 PMC3674908

[8] Cochrane Community. Living systematic reviews, https://community.cochrane.org/review-production/production-resources/living-systematic-reviews

[9] Elliott JH Turner T Clavisi O , et al. Living systematic reviews: an emerging opportunity to narrow the evidence-practice gap. PLoS Med 2014; 11(2): e1001603. 10.1371/journal.pmed.1001603 PMC3928029 24558353

[10] Cheyne S Fraile Navarro D Hill K , et al. Methods for living guidelines: early guidance based on practical experience. Paper 1: introduction. J Clin Epidemiol 2023; 155: 84–96. 36639038 10.1016/j.jclinepi.2022.12.024

[11] Akl EA El Khoury R Khamis AM , et al. The life and death of living systematic reviews: a methodological survey. J Clin Epidemiol 2023; 156: 11–21. 36764466 10.1016/j.jclinepi.2023.02.005

[12] Trials Tracker. https://covid19.trialstracker.net/

[13] COVID-NMA. https://covid-nma.com/

[14] Therapeutics and COVID-19: living guideline, 13 January 2023, https://www.who.int/publications/i/item/B09540

[15] Marshall C Sutton A O’Keefe H , et al. The systematic review toolbox, 2022, http://www.systematicreviewtools.com/10.1186/s13643-022-02122-z PMC9713957 36457048

[16] Marshall IJ Wallace BC. Toward systematic review automation: a practical guide to using machine learning tools in research synthesis. Syst Rev 2019; 8: 163. 31296265 10.1186/s13643-019-1074-9 PMC6621996

[17] Khalil H Ameen D Zarnegar A. Tools to support the automation of systematic reviews: a scoping review. J Clin Epidemiol 2022; 144: 22–42. 34896236 10.1016/j.jclinepi.2021.12.005

[18] Cowie K Rahmatullah A Hardy N , et al. Web-based software tools for systematic literature review in medicine: systematic search and feature analysis. JMIR Med Inform 2022; 10: e33219. 10.2196/33219 PMC9112080 35499859

[19] Harrison H Griffin SJ Kuhn I , et al. Software tools to support title and abstract screening for systematic reviews in healthcare: an evaluation. BMC Med Res Methodol 2020; 20: 7. 31931747 10.1186/s12874-020-0897-3 PMC6958795

[20] Schmidt L Olorisade BK McGuinness LA , et al. Data extraction methods for systematic review (semi)automation: a living systematic review. F1000Res 2021; 10: 401. 32724560 10.12688/f1000research.22781.1 PMC7338918

[21] Boesen K Hirt J Düblin P , et al. Rationale and design of the Cancer Immunotherapy Evidence Living (CIEL) library: a continuously updated clinical trial database of cancer immunotherapies. medRxiv [Preprint] 2024. DOI: 10.1101/2024.04.26.24306436. PMC13087519 42005618

[22] Tricco AC Lillie E Zarin W , et al. PRISMA extension for Scoping Reviews (PRISMA-ScR): checklist and explanation. Ann Intern Med 2018; 169: 467–473. 30178033 10.7326/M18-0850

[23] Citavi. https://www.citavi.com/en

[24] Rayyan. https://www.rayyan.ai/

[25] IBM. What is a relational database. n.d., https://www.ibm.com/topics/relational-databases

[26] Research Electronic Data Capture (REDCap). n.d., https://www.project-redcap.org/

[27] Genbank overview: what is Genbank? https://www.ncbi.nlm.nih.gov/genbank/

[28] Schmidt L Finnerty Mutlu AN Elmore R , et al. Data extraction methods for systematic review (semi)automation: update of a living systematic review. F1000res 2025; 10: 401. 10.12688/f1000research.51117.1 PMC8361807 34408850

[29] Rujano MA Boiten JW Ohmann C , et al. Sharing sensitive data in life sciences: an overview of centralized and federated approaches. Brief Bioinform 2024; 25: bbae262. 10.1093/bib/bbae262 PMC11151787 38836701

[30] Vivli. Center for global clinical research data. https://vivli.org/

[31] TrialResults-Centerorg. Searchable database of clinical trials results. https://www.trialresultscenter.org/evidenceTable/default_f.html

[32] Cucherat M. Trialresults-center: a web-based clinical trial results database providing dynamic systematic reviews and meta-analysis of clinical trials in cardiology. J Am Coll Cardiol 2010; 55(Suppl.10): A132E1239.

[33] Hetrick SE Parker AG Callahan P , et al. Evidence mapping: illustrating an emerging methodology to improve evidence-based practice in youth mental health. J Eval Clin Pract 2010; 16(6): 1025–1030. 20337833 10.1111/j.1365-2753.2008.01112.x

[34] De Silva S Bailey AP Parker AG , et al. Open-access evidence database of controlled trials and systematic reviews in youth mental health. Early Interv Psychiatry 2019; 3: 474–477. 10.1111/eip.12423 28488387

[35] Evidence Finder. https://orygen.org.au/Training/Evidence-Finder

[36] Cochrane. https://covid-19.cochrane.org/

[37] COVID-evidence. https://covid-evidence.org/

[38] EPPI centre COVID-19 living map of the evidence. https://eppi.ioe.ac.uk/cms/Projects/NIHRPolicyReviewsFacility/COVID-19Alivingmapoftheevidence/tabid/3931/Default.aspx

[39] MetaEvidence breast cancer. http://www.metaevidence.org/default0.aspx

[40] Meta Evidence COVID-19. http://www.metaevidence.org/default0.aspx

[41] MetalO. http://www.metaevidence.org/default0.aspx

[42] MetaPreg. http://www.metaevidence.org/default0.aspx

[43] Infectious Disease Data Observatory. A living systematic review of registered COVID-19 trials, https://www.iddo.org/covid-19/live-systematic-review-trials

[44] Worldwide Antimalarial Resistance Network (WWARN). Clinical trials publication library. Malaria Data Inventory, https://www.wwarn.org/tools-resources/literature-reviews/wwarn-clinical-trials-publication-library

[45] Worldwide Antimalarial Resistance Network (WWARN). Accessing data. Malaria Data Inventory, n.d., https://www.iddo.org/wwarn/accessing-data

[46] Takata J Sondo P Humphreys GS , et al. The worldwide antimalarial resistance network clinical trials publication library: a live, open-access database of plasmodium treatment efficacy trials. Am J Trop Med Hyg 2020; 103(1): 359–368. 32431267 10.4269/ajtmh.19-0706 PMC7356478

[47] Probst P Hüttner FJ Meydan Ö , et al. Evidence map of pancreatic surgery: protocol for a living systematic review and meta-analysis. BMJ Open 2019; 9: e032353. 10.1136/bmjopen-2019-032353 PMC6773280 31575583

[48] Probst P Hüttner FJ Meydan Ö , et al. Evidence map of pancreatic surgery-A living systematic review with meta-analyses by the International Study Group of Pancreatic Surgery (ISGPS). Surgery 2021; 170(5): 1517–1524. 34187695 10.1016/j.surg.2021.04.023

[49] Evidence Map. Living evidence map of pancreatic surgery, https://map.eviglance.com/maps/8?view=map

[50] Canham S Ohmann C. A metadata schema for data objects in clinical research. Trials 2016; 17: 557. 27881150 10.1186/s13063-016-1686-5 PMC5122021

[51] Sim I Owens DK Lavori PW , et al. Electronic trial banks: a complementary method for reporting randomized trials. Med Decis Making 2000; 20(4): 440–450. 11059477 10.1177/0272989X0002000408

[52] Sim I Carini S Olasov B , et al. Trial bank publishing: phase I results. Stud Health Technol Inform 2004; 107(pt.2): 1476–1480. 15361060

[53] Sim I Detmer DE. Beyond trial registration: a global trial bank for clinical trial reporting. PLoS Med 2005; 2(11): e365. 10.1371/journal.pmed.0020365 PMC1255763 16220999

[54] The Trial Bank Project. https://grantome.com/grant/NIH/R01-LM006780-10

[55] De Angelis C Drazen JM Frizelle FA , et al. Clinical trial registration: a statement from the International Committee of Medical Journal Editors. CMAJ 2004; 171: 606–607. 15367465 10.1503/cmaj.1041281 PMC516198

[56] Goldacre B DeVito NJ Heneghan C , et al. Compliance with requirement to report results on the EU Clinical Trials Register: cohort study and web resource. BMJ 2018; 362: k3218. 10.1136/bmj.k3218 PMC6134801 30209058

[57] Wieseler B. Beyond journal publications – a new format for the publication of clinical trials. Z Evid Fortbild Qual Gesundhwes 2017; 120: 3–8. 28284364 10.1016/j.zefq.2016.11.003

[58] Dunn AG Bourgeois FT. Is it time for computable evidence synthesis? J Am Med Inform Assoc 2020; 27: 972–975. 32337600 10.1093/jamia/ocaa035 PMC7309243

[59] Critical Path Institute. Tools and platforms, https://c-path.org/tools-platforms/

[60] Hubscher E Chennakrishnaiah S Forsythe A. HTA102 machine-learning technology assisted curated reference libraries as an approach for rapid global value dossier updates to support living health technology assessment: a case study in Triple Refractory Multiple Myeloma (TRMM). Value Health 2022; 25: S316.

[61] Critical Path Institute. Critical Path for Alzheimer’s Disease, https://c-path.org/programs/cpad/

[62] LaRocca NG Hudson LD Rudick R , et al. The MSOAC approach to developing performance outcomes to measure and monitor multiple sclerosis disability. Mult Scler 2018; 24(11): 1469–1484. 28799444 10.1177/1352458517723718 PMC6174619

[63] Critical Path Institute. Critical path for Parkinson’s, https://c-path.org/program/critical-path-for-parkinsons/

[64] Critical Path Institute. TB-Platform for Aggregation of Clinical TB Studies (TB-PACTS), https://c-path.org/tools-platforms/tb-pacts/

[65] TrialStreamer. https://trialstreamer.ieai.robotreviewer.net/

[66] Wellcome Trust. https://wellcome.org/news/evidence-synthesis-infrastructure-collaborative

[67] Cristea IA Cahan EM Ioannidis JPA . Stealth research: lack of peer-reviewed evidence from healthcare unicorns. Eur J Clin Invest 2019; 49(4): e13072. 10.1111/eci.13072 30690709

[68] Cytel. LiveSLR. https://www.cytel.com/live-slr

[69] Liu J Verhoek A Thorlund K , et al. HTA35 should Health Technology Assessment (HTA) bodies utilize living HTA tools? Validation of LiveSLR® and LiveNMA™ tools using ICER’S class review in Relapsed Refractory Multiple Myeloma (RRMM). Value Health 2022; 25: S302–S303.

