# Supplementary material for: Topic-specific living databases of clinical trials: A scoping review of public databases
Source: Clin Trials. 2026 Jan 5;23(2):198–209. doi: 10.1177/17407745251400635 (PMC13050396; doi:10.1177/17407745251400635)
Supplement: sj-pdf-1-ctj-10.1177_17407745251400635 – Supplemental material for Topic-specific living databases of clinical trials: A scoping review of public databases [file sj-pdf-1-ctj-10.1177_17407745251400635.pdf]

Topic-specific living databases of clinical trials: A scoping review of public databases

Boesen et al.

Appendix

S1. Clinical trial databases known before scoping review

S2. Cold searches to identify seed references and key words

S3. Search string (PubMed)

S4. Search string (Embase)

S5. PRISMA 2020 flowchart of the literature search and selection process

S6. List of excluded reports with reasons at full text screening stage (n=14)

S7. Screenshots of trial presentations

S1. Clinical trial databases known before the scoping review

**Platform**	**Funder**	**Type of platform**	**Comments**
Cochrane COVID-19 study register<br>https://covid-19.cochrane.org/	Cochrane Collaboration	Database of COVID studies, including but not restricted to RCTs	-
COVID-evidence https://covid-evidence.org/	Swiss National Science Foundation	Database of COVID RCTs	Not updated since 31/12/2022
COVID-NMA initiative https://covid-nma.com/	CRESS and Cochrane France	Live mapping and network meta-analyses of COVID trials	-
COVID 19 Trial Tracker<br>https://covid19.trialstracker.net/	CEBM, Oxford University	Database of COVID RCTs	-
EPPI Centre Covid-19 Living Map of the Evidence<br>https://eppi.ioe.ac.uk/eppi-vis/Review/Index/7	EPPI Centre	Database of COVID studies, including but not restricted to RCTs	-
MetaEvidence COVID<br>http://www.metaevidence.org/default0.aspx	Hospices Civils de Lyon	Database of COVID RCTs	No longer updated
MetaEvidence breast cancer<br>http://www.metaevidence.org/default0.aspx	Hospices Civils de Lyon	Database of breast cancer treatments	-
MetaIO<br>http://www.metaevidence.org/default0.aspx	Hospices Civils de Lyon	Database of immuno-oncology trials	-
PragMeta*<br>https://pragmeta.org/	Swiss National Science Foundation	Database of pragmatic trials	Not continuously updated

*Not qualifying as a 'continuously updated field-specific databases'; either not continuously updated or not clinical trial or field specific, or unable to assess the used methods (no descriptions, or offline).

S2. Cold searches to identify seed references and key words

Source	Search string	No. hits	Potential relevant hits
**Pubmed**	“living library”	8	0
	“living review”[a]	67	0
	“living systematic review”[b]	276	1 (Evidence map)
	“Living evidence map”	4	1 (Evidence map)
	“Living platform”	6	0
	“Living database”	11	1 (Trial streamer)
	“Clinical trials platform”	82	0
	“interactive database”[c]	258	0
**PROSPERO**	“Living library”	1	0
	“Interactive database”	0	0
	“Clinical trials platform”	80	0
**medRxiv**	“Living library”	0	0
	“Living review”	84	0
	“Evidence map”	49	1 (Trialstreamer)
	“Database clinical trials”	12	0
**ArXiv**	“information retrieval” AND “medical” AND ”trial” (field codes)	9	1 (TrialStreamer)
**OSF**	“Living library”	0	
	“Living database” (project)	6	1 (Covid evidence)
	“Evidence map” (project)	37	0
	“Clinical trials platform”	2	0
**Google**	“Living library clinical trials”, “library clinical trials”, “living platform clinical trials”	Infinitive	3 (Atlas Edu, IDDO, WWARN)
**Other sources**	-	-	1 (Evidence Finder)

a) Mostly regular “paper based” living systematic reviews.
b) Mostly regular systematic reviews, majority about COVID.
c) Many hits related to genomic databases, such as https://doi.org/10.1093/nar/gkz884 (database for snoDNA); https://doi.org/10.3389/fgene.2019.00262 (database for human single nucleotide polymorphism SNP).

S3. Search string (PubMed)

MEDLINE/PubMed

Search Feb 1, 2023:

#	Entry	Hits
1	(database*[title] OR librar*[title] OR platform*[title] OR hub[title] OR repositor*[title] OR collection*[title] OR overview*[title] OR archive*[title] OR index*[title] OR bibliograph*[title] OR catalogue*[title] OR system[title] OR storage*[title] OR data*[title] OR retrieval*[title] OR inventor*[title] OR portal[title]) AND (living[title] OR up-to-date[title] OR updat*[title] OR continuous*[title] OR accumulat*[title] OR dynamic[title] OR interactive[title] OR digital[title]) AND (rct[tiab] OR trial[tiab] OR "clinical study"[tiab] OR "Clinical Studies as Topic"[MeSH])	1,079

S4. Search string (Embase)

Embase/Elsevier

Search Feb 1, 2023:

#	Entry	Hits
1	(database*:ti OR librar*:ti OR platform*:ti OR hub:ti OR repositor*:ti OR collection*:ti OR overview*:ti OR archive*:ti OR index*:ti OR bibliograph*:ti OR catalogue*:ti OR system:ti OR storage*:ti OR data*:ti OR retrieval*:ti OR inventor*:ti OR portal:ti) AND (living:ti OR 'up to date':ti OR updat*:ti OR continuous*:ti OR accumulat*:ti OR dynamic:ti OR interactive:ti OR digital:ti) AND (rct:ti,ab OR trial:ti,ab OR 'clinical study':ti,ab OR 'clinical study'/de)	1,507
2	#1 AND [embase]/lim NOT ([embase]/lim AND [medline]/lim)	725

S5. PRISMA 2020 flowchart of the literature search and selection process

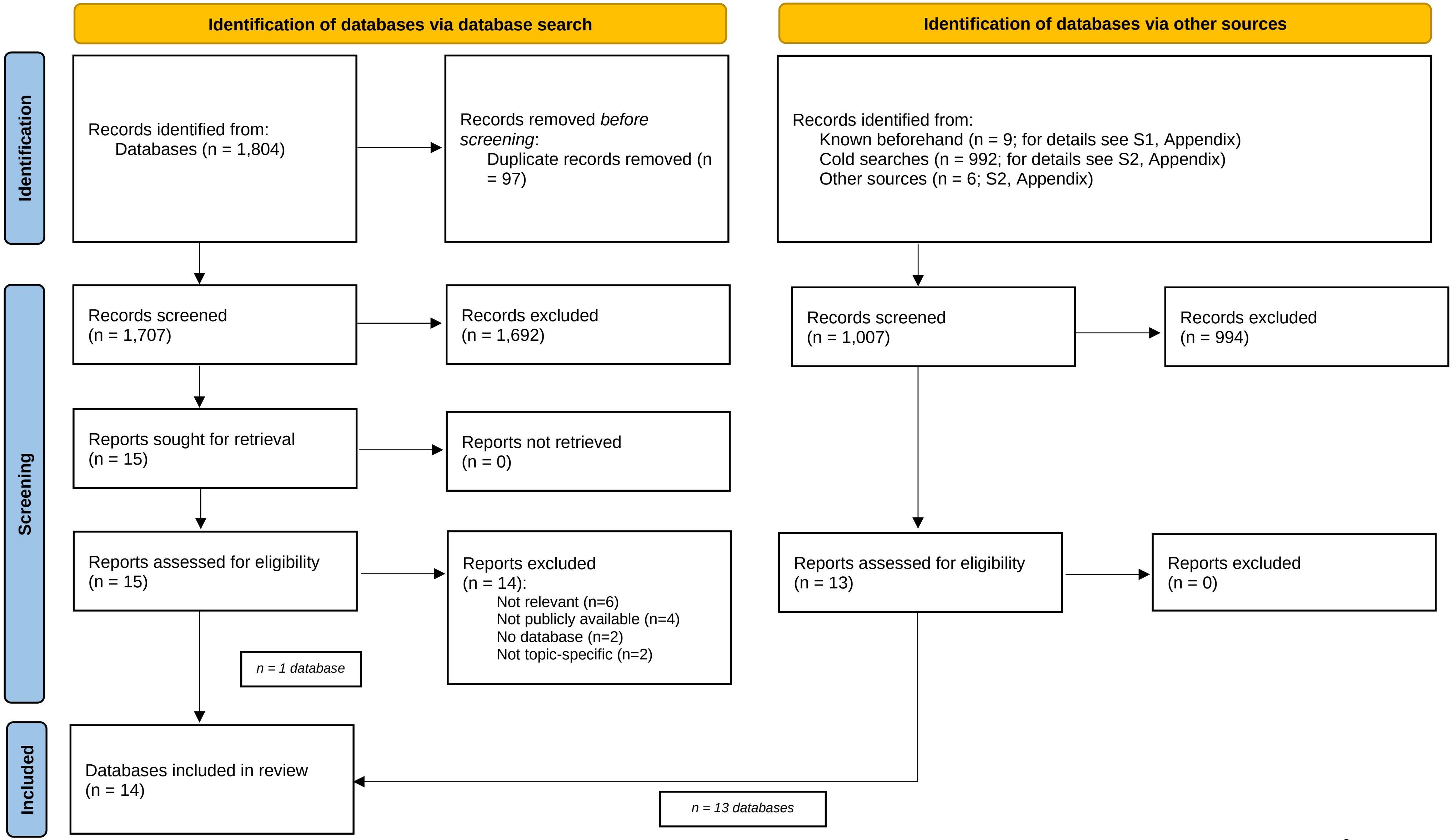

S6. List of excluded reports with reasons at full text screening stage (n=14)

Excluded (n=14)

1. Peyser et al (2022). Digital platforms for clinical trials: The Eureka experience. https://doi.org/10.1016/j.cct.2022.106710.

Reason: **Not relevant**. Eureka is a platform aimed to facilitate trial conduct; it is not a topic-specific live database.

2. Renfro et al (2013). **Mining the ACCENT** database**: a review and update. https://doi.org/**10.3978/j.issn.2304-3865.2013.03.05.

Reason: **Not publicly available**. The ACCENT database is a large database of colon cancer trials including individual patient data for more than 35.000 patients. The database is not publicly available and it is uncertain whether it could be characterised as a continuously updated database. https://doi.org/10.1093/jnci/djab123.

3. Bronsert et al (2014). The freiburg bio-databanking combines automaticly updated pathological and clinical information with research data. https://doi.org/10.1159/000360634.

Reason: **Not publicily available**. According to the abstract it it also uncertain whether it included relevant clinical trial information. Searches on Google (Freiburg bio-databank Bronsert) did not help identify the database.

4. Broglio et al (2013). **A meta-analysis** platform **for the continuous updating of knowledge regarding treatment regimens for hepatitis C virus (HCV) infection. https://doi.org/10.1002/hep.26882.**

Reason: Not publicly available. According to the abstract also uncertain whether it is continuously updated. Further information was found in a full article (searching for the first author Broglio KR) https://pubmed.ncbi.nlm.nih.gov/25825840/. The platform was based on a standard systematic review of the literature, thus it would not be considered eligible.

5. Carles et al. (2018). PKIDB: A Curated, Annotated and Updated Database of Protein Kinase Inhibitors in Clinical Trials. https://doi.org/10.3390/molecules23040908.

Reason: Not relevant. A publicly available, continously updated database on protein-kinase inhibitors used in clinical trials. The database is nicely done, data is downloadable, yet the topic is focusing on the drugs, not the actual trials. https://www.icoa.fr/pkidb/.

6. Kang et al. (2019). DRAMP 2.0, an updated data repository of antimicrobial peptides. https://doi.org/10.1038/s41597-019-0154-y.

Reason: Not relevant. A publicly available, continuously updated database of animicrobial peptides. Similarly to the PKIBD, it is nicely done, is open, but the focus is on the library of peptides, not on the clinical trials. http://dramp.cpu-bioinfor.org/.

7. Jung et al. (2021). **Real-time automatically updated data on healthcare (ROOT): An innovative and automated data collection system. https://doi.org/**10.21037/tlcr-21-531.

Reason: Not relevant. Not database of clinical trials but of individual patient data and tumor biology and histopathology.

8. Jung et al. Successful Development of Realtime Automatically Updated Data Warehouse in Health Care (ROOT-S). https://doi.org/10.1016/j.jtho.2019.08.659.

Reason: Not relevant. Same sources as above.

9. Juul et al. (2021). Interventions for treatment of COVID-19: a protocol for a living systematic review with network meta-analysis including individual patient data (The LIVING Project). https://doi.org/10.1186/s13643-020-01371-0.

Reason: No database. Traditional systematic review, not a continuously updated database.

10. Korang et al. (2021). Vaccines to prevent COVID-19: a protocol for a living systematic review with network meta-analysis including individual patient data (The LIVING VACCINE Project). https://doi.org/10.1186/s13643-020-01516-1.

Reason: No database. Same as above; not a continuously updated database.

11. Hubscher et al. (2022). Machine-Learning Technology Assisted Curated Reference Libraries as an Approach for Rapid Global Value Dossier Updates to Support Living Health Technology Assessment: A Case Study in Triple Refractory Multiple Myeloma (TRMM). https://doi.org/10.1016/j.jval.2022.09.1561.

Reason: Not publicly available. Abstract only, not able to identify full database searching for first author on Google Scholar.

12. Marshall et al. (2020). Trialstreamer: A living, automatically updated database of clinical trial reports. https://doi.org/10.1093/jamia/ocaa163.

Reason: Not topic specific. Generic large-scale database of clinical trials, not focusing on particular topics.

13. Surian et al. (2021). The automation of relevant trial registration screening for systematic review updates: an evaluation study on a large dataset of ClinicalTrials.gov registrations. https://doi.org/10.1186/s12874-021-01485-6.

Reason: Not topic specific. Method's paper describing how new systematic reviews should be updated based on screening of clinical trial registries. Published by the same group behind trial2rev (https://pubmed.ncbi.nlm.nih.gov/31984340/).

14. Stevenson et al. (2018). Etarget: A digital science solution to integrate clinical and genomic data for the manchester molecular tumour board (MTB). https://doi.org/10.1136/esmoopen-2018-EACR25.580.

Reason: Not relevant. Based on the website for the team behind eTarget (https://digitalecmt.org/2019/04/nature-medicine-publication-target-trial-results-etarget/), it pertains to individual patient data, not clinical trials. Also it doesn't seem like a publicly available database.

S7. Screenshots of trial presentations

Cochrane COVID-19 study register

STUDY RECORD

MURAKAMI, 21 OCT 2023 | First Published: 2024 Feb 4 | Updated Date: 2024 Feb 4

Perceptions and responses to COVID-19 through wastewater surveillance information and online search behavior: A randomized controlled trial

Study Type	Study Aim	Study Design	Intervention Assignment
Interventional	Epidemiology	Parallel/Crossover	Randomised

REFERENCE RECORD

Perceptions and responses to COVID-19 through wastewater surveillance information and online search behavior: A randomized controlled trial

Murakami, M.; Nomura, S.; Ando, H.; Kitajima,,M...
First Published | ↗ Medrxiv (https://www.medrxiv.org/content/early/2023/10/21/2023.10.20.23297297)
Unpublished article (preprint)
Report Results

Wastewater surveillance is anticipated to be a representative and timely method to assess infectious disease status; however, its influence on public perception and behavior remains unclear. Therefore, in this study, we used a randomized controlled trial to analyze the influence of wastewater surveillance-based information on understanding of, interest in, relief regarding, preventive behavioral intention against, and subsequent online search behavior related to coronavirus disease 2019 (COVID-19). Valid responses were obtained from 1,000 individuals in both control and intervention groups from Yahoo crowdsourcing users aged [≥]18 years in Japan. This survey was conducted from August 4 to August 7, 2023, just before the common Japanese tradition of returning to hometowns. The questionnaire not only collected personal attributes but also gauged responses to COVID-19 information. This information highlighted the early detection capabilities and representativeness of wastewater surveillance compared with sentinel surveillance at medical institutions. At one-week post-survey, we obtained the survey participants' online search history for key words such as "bullet train," "highway," "airplane," and "wastewater." The findings showed no significant differences between the two groups in terms of COVID-19 interest or preventive behavior before information provision, verifying the effectiveness of participant randomization. Wastewater surveillance-based information did not notably elevate understanding or specific intentions regarding COVID-19, such as wearing masks and receiving vaccination. However, it significantly increased interest in, relief concerning the infection status, and general preventive behavioral intentions. Heightened interest and general preventive intentions did not depend on prior interest or behavior. However, those who previously engaged in preventive behavior or who were less interested in COVID-19 exhibited more relief after exposure to wastewater surveillance-based information. Furthermore, this information could slightly influence online searches related to return travel modes, such as highways. In conclusion, information from wastewater surveillance effectively shapes individual perceptions of and responses to infections. [Preprints are preliminary reports of work that have not been peer reviewed. Refer to the original preprint or preprint server for specific information about the individual preprint.]

COVID Evidence

- **Bringing Optimised COVID-19 Vaccine Schedules To ImmunoCompromised Populations (BOOST-IC): an Adaptive Randomised Controlled Clinical Trial**

  NCT05556720 Location: Australia (Australia/Oceania) Status: Not Yet Recruiting Registration Date: 2022-09-27 Start Date: 2022-10-01 Estimated End Date: 2025-12-31

  Brief summary: Despite the greater risk of adverse COVID-19 outcomes, antibody and cell-mediated immune responses to COVID-19 vaccines vary amongst immunocompromised (IC) people and are poorly defined. IC hosts were largely excluded from the COVID-19 vaccine registration trials, though many countries...

  @clinicaltrials.gov

COVID NMA initiative

Treatment (per arm)	Sample size	Severity at enrollment	Sponsor/Funder	Reg. number
(1) Nirmatrelvir + ritonavir *vs* (2) Nirmatrelvir + ritonavir *vs* (3) Placebo	900	Patients recovered from covid	Kanecia Obie Zimmerman	NCT05595369
(1) Imidazolyl ethanamide pentandioic acid *vs* (2) Placebo	233	Mild	Valenta Pharm JSC	NCT05216497
(1) Isotretinoin *vs* (2) Isotretinoin *vs* (3) Standard of care	100000	Critical	Kafrelsheikh University	NCT04353180
(1) Interferon gamma *vs* (2) Standard of care	630	Healthy volunteers	SPP Pharmaclon Ltd.	NCT05054114
(1) Fluticasone *vs* (2) Standard of care	500	Mild	University of Medicine and Pharmacy at Ho Chi Minh City	NCT05054322
(1) Baricitinib *vs* (2) Placebo	480	Moderate/severe	Incepta Pharmaceuticals Ltd	NCT05056558
(1) Platelet rich plasma *vs* (2) Placebo	30	Patients recovered from covid	Stanford University	NCT04406584
(1) Masitinib *vs* (2) Masitinib *vs* (3) Masitinib *vs* (4) Placebo	78	Mild/moderate	AB Science	NCT05047783
(1) Isotretinoin + tamoxifen *vs* (2) Isotretinoin + tamoxifen	160	Severe/critical	Kafrelsheikh University	NCT04389580
(1) Placebo	400	Healthy volunteers	Chumakov Federal Scientific Center for Research and Development of Immune-and-Biological Products	NCT05046548

COVID-19 Trials Tracker

index	trial_id	registry	registration_date	start_date	retrospective_registration	sponsor
0	ChiCTR2000029953	ChiCTR	2020-02-17	2020-02-01	True	Zhongnan Hospital of Wu[illegible]
1	ChiCTR2000029949	ChiCTR	2020-02-16	2020-02-16	False	Zhongnan Hospital of Wu[illegible]
2	ChiCTR2000029947	ChiCTR	2020-02-16	2020-03-01	False	Longhua Hospital Affiliate[illegible] University of Traditional C[illegible]

EPPI Centre Covid-19 Living Map of the Evidence

Tocilizumab in patients admitted to hospital with COVID-19 (RECOVERY): a randomised, controlled, open-label, platform trial

Abstract: <h2>Summary</h2><h3>Background</h3> In this study, we aimed to evaluate the effects of tocilizumab in adult patients admitted to hospital with COVID-19 with both hypoxia and systemic inflammation. <h3>Methods</h3> This randomised, controlled, open-label, platform trial (Randomised Evaluation of COVID-19 Therapy [RECOVERY]), is assessing several possible treatments in patients hospitalised with COVID-19 in the UK. Those trial participants with hypoxia (oxygen saturation <92% on air or requiring oxygen therapy) and evidence of systemic inflammation (C-reactive protein ≥75 mg/L) were eligible for random assignment in a 1:1 ratio to usual standard of care alone versus usual standard of care plus tocilizumab at a dose of 400 mg–800 mg (depending on weight) given intravenously. A second dose could be given 12–24 h later if the patient's condition had not improved. The primary outcome was 28-day mortality, assessed in the intention-to-treat population. The trial is registered with ISRCTN (50189673) and ClinicalTrials.gov (NCT04381936). <h3>Findings</h3> Between April 23, 2020, and Jan 24, 2021, 4116 adults of 21 550 patients enrolled into the RECOVERY trial were included in the assessment of tocilizumab, including 3385 (82%) patients receiving systemic corticosteroids. Overall, 621 (31%) of the 2022 patients allocated tocilizumab and 729 (35%) of the 2094 patients allocated to usual care died within 28 days (rate ratio 0·85; 95% CI 0·76–0·94; p=0·0028). Consistent results were seen in all prespecified subgroups of patients, including those receiving systemic corticosteroids. Patients allocated to tocilizumab were more likely to be discharged from hospital within 28 days (57% <i>vs</i> 50%; rate ratio 1·22; 1·12–1·33; p<0·0001). Among those not receiving invasive mechanical ventilation at baseline, patients allocated tocilizumab were less likely to reach the composite endpoint of invasive mechanical ventilation or death (35% <i>vs</i> 42%; risk ratio 0·84; 95% CI 0·77–0·92; p<0·0001). <h3>Interpretation</h3> In hospitalised COVID-19 patients with hypoxia and systemic inflammation, tocilizumab improved survival and other clinical outcomes. These benefits were seen regardless of the amount of respiratory support and were additional to the benefits of systemic corticosteroids. <h3>Funding</h3> UK Research and Innovation (Medical Research Council) and National Institute of Health Research.

Author(s) ;

Journal The Lancet

Full record

Coding Report:

Topic

- Treatment evaluation

Version

- All versions

Previous versions 81 to 100

- Version 90 - 30th January 2022

Evidence Finder

COGNITIVE BIAS MODIFICATION FOR SOCIAL ANXIETY: THE DIFFERENTIAL IMPACT OF MODIFYING ATTENTIONAL AND/OR INTERPRETATION BIAS

Yeung, E. S., Sharpe, L.

Cognitive Therapy and Research., 43 : 781 - 791

- Year: 2019
- Problem: Social phobia (social anxiety disorder)
- Type: Randomised controlled trials
- Stage: At risk (indicated or selected prevention)

Evidence Map

Superiority of Step-up Approach vs Open Necrosectomy in Long-term Follow-up of Patients With Necrotizing Pancreatitis

Hollemans et al.
Gastroenterology.
2019;156(4):1016-1026

Conclusion

In an analysis of long-term outcomes of trial participants, we found the step-up approach for necrotizing pancreatitis to be superior to open necrosectomy, without increased risk of reinterventions.

Overall risk of bias judgement

Some concerns

Funding

Non-industry funded

IDDO

The effect of zinc supplementation on inflammatory responses and total blood cells counts of patients who infected with COVID-19

Country
Iran (Islamic Republic of)

Unique trial registry identifier
IRCT20210204050238N1

Date the clinical trial record was first registered with the source clinical trial registry
2021-03-02

Additional unique registration identifiers if the trial was registered with multiple clinical trial registries
-

Short hand reference or acronym assigned to the study. E.g. NOSO-COR; SOLID-C19; SOLIDARITY
-

Source
Iranian Registry of Clinical Trials

Recruitment Status
Completed

MetaEvidence breast cancer

I-SPY2, 2020 randomized controlled trial   Risk of bias high

JAMA Oncol 2020 May - Cancer 2014; 120:773 - JAMA Oncol 2020 Sep 1;6(9):1355-1362. - NCT01042379

Treatments	Patients	Method
pembrolizumab plus paclitxel followed by doxorubicin plus cyclophosphamide (n=69)<br>200mg intravenous pembrolizumab every 3weeks for 4 cycles plus standard NACT: 80 mg/m2 intravenous paclitaxel weekly for 12 weeks, followed by 4 cycles of 60 mg/m2 doxorubicin plus 600 mg/m2 intravenous cyclophosphamide every 2 to 3 weeks (AC).<br>vs.<br>paclitaxel followed by doxorubicin plus cyclophosphamide (n=181)<br>standard NACT: 80 mg/m2 intravenous paclitaxel weekly for 12 weeks, followed by 4 cycles of 60 mg/m2 doxorubicin plus 600 mg/m2 intravenous cyclophosphamide every 2 to 3 weeks (AC).<br>If patients had an infusion reaction despite corticosteroid premedications, switching to nab-paclitaxel was allowed.	Patients with ERBB2 (formerly HER2)-negative breast cance, women treated with neoadjuvant for early-stage breast cancer (stage II or III)<br>stage II or III breast cancer and primary tumors larger than 2.5 cmby clinical examination or larger than 2.0cmby imaging	Blindness: open label<br>inclusion period: November 26, 2015, and November 5, 2016,<br>Sample size: 69/181<br>Primary endpoint: pCR<br>FU duration: 33.6 vs 42<br>Sponsor: Quantum Leap Healthcare Collaborative<br>P2 /two sided 0,05; BAYESIAN STUDY

! the study did not meet its primary endpoint pCR

exploratory study (phase 2)

trial #5132 MA 235

Meta-Evidence COVID

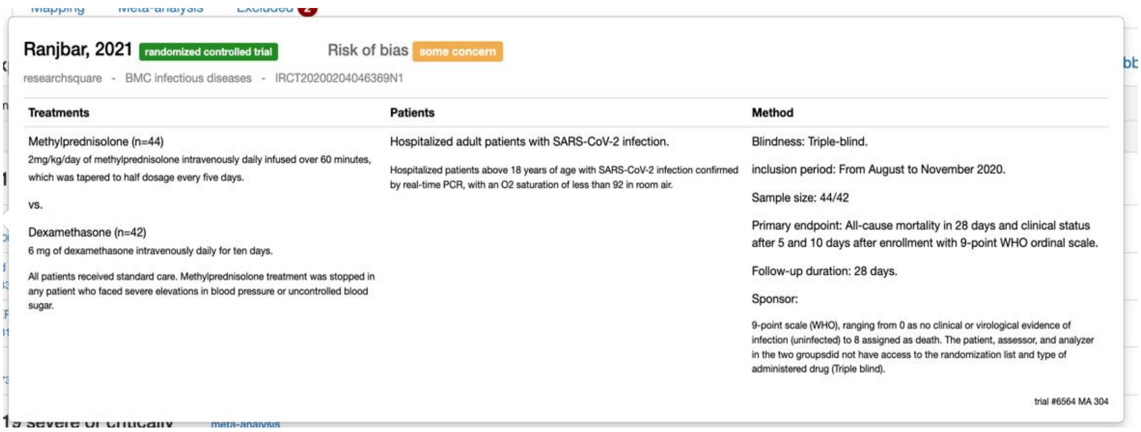

Ranjbar, 2021 randomized controlled trial — Risk of bias: some concern

researchsquare - BMC infectious diseases - IRCT20200204046369N1

Treatments	Patients	Method
Methylprednisolone (n=44)<br>2mg/kg/day of methylprednisolone intravenously daily infused over 60 minutes, which was tapered to half dosage every five days.<br>vs.<br>Dexamethasone (n=42)<br>6 mg of dexamethasone intravenously daily for ten days.<br>All patients received standard care. Methylprednisolone treatment was stopped in any patient who faced severe elevations in blood pressure or uncontrolled blood sugar.	Hospitalized adult patients with SARS-CoV-2 infection.<br>Hospitalized patients above 18 years of age with SARS-CoV-2 infection confirmed by real-time PCR, with an O2 saturation of less than 92 in room air.	Blindness: Triple-blind.<br>inclusion period: From August to November 2020.<br>Sample size: 44/42<br>Primary endpoint: All-cause mortality in 28 days and clinical status after 5 and 10 days after enrollment with 9-point WHO ordinal scale.<br>Follow-up duration: 28 days.<br>Sponsor:<br>9-point scale (WHO), ranging from 0 as no clinical or virological evidence of infection (uninfected) to 8 assigned as death. The patient, assessor, and analyzer in the two groupsdid not have access to the randomization list and type of administered drug (Triple blind).

trial #6564 MA 304

MetaIO

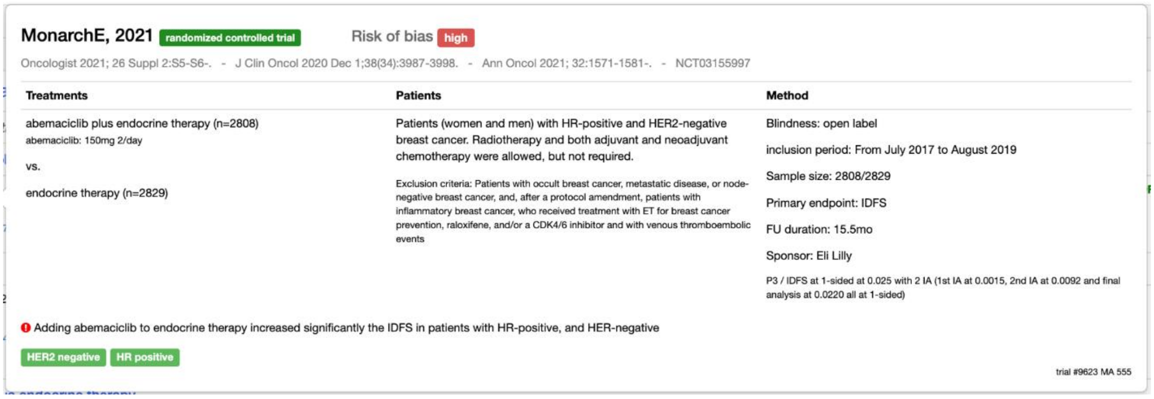

MonarchE, 2021 randomized controlled trial — Risk of bias: high

Oncologist 2021; 26 Suppl 2:S5-S6-. - J Clin Oncol 2020 Dec 1;38(34):3987-3998. - Ann Oncol 2021; 32:1571-1581-. - NCT03155997

Treatments	Patients	Method
abemaciclib plus endocrine therapy (n=2808)<br>abemaciclib: 150mg 2/day<br>vs.<br>endocrine therapy (n=2829)	Patients (women and men) with HR-positive and HER2-negative breast cancer. Radiotherapy and both adjuvant and neoadjuvant chemotherapy were allowed, but not required.<br>Exclusion criteria: Patients with occult breast cancer, metastatic disease, or node-negative breast cancer, and, after a protocol amendment, patients with inflammatory breast cancer, who received treatment with ET for breast cancer prevention, raloxifene, and/or a CDK4/6 inhibitor and with venous thromboembolic events	Blindness: open label<br>inclusion period: From July 2017 to August 2019<br>Sample size: 2808/2829<br>Primary endpoint: IDFS<br>FU duration: 15.5mo<br>Sponsor: Eli Lilly<br>P3 / IDFS at 1-sided at 0.025 with 2 IA (1st IA at 0.0015, 2nd IA at 0.0092 and final analysis at 0.0220 all at 1-sided)

Adding abemaciclib to endocrine therapy increased significantly the IDFS in patients with HR-positive, and HER-negative

HER2 negative HR positive

trial #9623 MA 555

MetaPreg

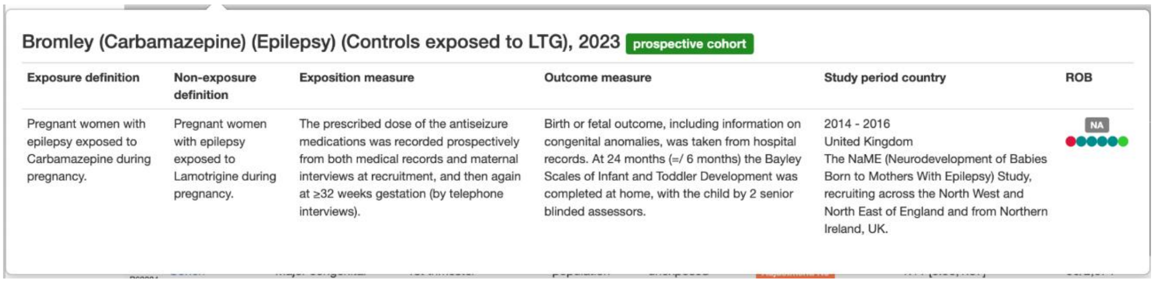

Bromley (Carbamazepine) (Epilepsy) (Controls exposed to LTG), 2023 prospective cohort

Exposure definition	Non-exposure definition	Exposition measure	Outcome measure	Study period country	ROB
Pregnant women with epilepsy exposed to Carbamazepine during pregnancy.	Pregnant women with epilepsy exposed to Lamotrigine during pregnancy.	The prescribed dose of the antiseizure medications was recorded prospectively from both medical records and maternal interviews at recruitment, and then again at ≥32 weeks gestation (by telephone interviews).	Birth or fetal outcome, including information on congenital anomalies, was taken from hospital records. At 24 months (=/ 6 months) the Bayley Scales of Infant and Toddler Development was completed at home, with the child by 2 senior blinded assessors.	2014 - 2016<br>United Kingdom<br>The NaME (Neurodevelopment of Babies Born to Mothers With Epilepsy) Study, recruiting across the North West and North East of England and from Northern Ireland, UK.	NA

TrialsResultsCenter

Not working properly.

WWARN

	A	B	C	D	E	F	G	H	I	J	K	L	M	N
1	Nu	Repeat Instru	Repeat Insta	Authors of th	Year of Publi	Title of Publi	Journal of Pu	Journal Volu	Issue Numbe	Pages of Arti	Clinical Trials	Region of Stu	Country of St	Language of
2	12936-022-04143-4			Pembet Sing	2022	Prevalence o	Malaria journ	21	1	137	ACTRN12616	Africa	Congo (Demo	English
3	12	Treatment	1											
4	12	Treatment	2											
5	12	Site	1											
6	12936-022-04350-z			Assefa, A.; H	2022	Therapeutic	Malaria journ	21	359	Jan/13	Not Provided	Africa	Ethiopia (ETH	English
7	12	Treatment	1											
8	12	Treatment	2											
9	12	Treatment	3											
10	12	Treatment	4											
11	12	Site	1											
12	12	Site	2											
13	12936-022-04380-7			Hussein, Mol	2022	Therapeutic	Malaria journ	21	351	Jan/13	Not Provided	Africa	Ethiopia (ETH	English
14	12	Treatment	1											
